# Harpagide attenuates cerebral ischemic injury by modulating mitochondrial calcium homeostasis associated with the AMPK–MCU axis

**DOI:** 10.1038/s41598-026-53322-9

**Published:** 2026-06-01

**Authors:** Ke Wang, Huai-yu Liu, Yue Wang, Gen-zheng Su, Jin-lai Gao, Yan-Ping Wang, Zhi He

**Affiliations:** 1 Department of Pharmacology, College of Medicine, Jiaxing University, Jiaxing, 314001 People’s Republic of China; 2 College of Medicine and Health Sciences, China Three Gorges University, Yichang, 443002 People’s Republic of China; 3 Xinmai Pharmaceutical Co., Ltd., Beijing, 100195 People’s Republic of China; 4 College of Life Science and Medicine, Zhejiang Sci-Tech University, Hangzhou, 310018 People’s Republic of China; 5 JiaXing Key Laboratory of Fundamental and Clinical Research on Cerebral Protection in Acute Ischemic Cerebrovascular Disease(A), Jiaxing, 314001 People’s Republic of China

**Keywords:** Harpagide, Ischemic stroke, Mitochondrial calcium homeostasis, MCU, AMPK, Cell biology, Diseases, Neurology, Neuroscience

## Abstract

Mitochondrial calcium homeostasis may offer therapeutic benefits for ischemic stroke. Harpagide has been shown to inhibit mitochondrial calcium uptake, but the mechanism remains unclear. In this study, a male ICR mouse model of permanent middle cerebral artery occlusion (pMCAO)-induced focal cerebral ischemia and PC12 and SH-SY5Y cells exposed to oxygen–glucose deprivation (OGD) were used. Apoptosis, lactate dehydrogenase (LDH), superoxide dismutase (SOD), malondialdehyde (MDA), and the ATP levels were measured to assess the neuroprotective effects of harpagide. DCFH-DA, JC-1, and Rhod-2A probes were used to analyze the mitochondrial function. RT-qPCR and western blot were used to determine the mitochondrial calcium uniporter (MCU), the apoptosis-inducing factor (AIF), Endo G, Cyt C, Caspase-3, and AMPK–MCU phosphorylation expression levels. Additionally, Longa scoring and triphenyl tetrazolium chloride (TTC) staining were used. Transmission electron microscopy (TEM) was used to examine the mitochondrial ultrastructural changes. An immunohistochemical analysis was conducted to detect the MCU expression in the temporal cortex. Harpagide attenuated OGD-induced cytotoxicity, as evidenced by reduced lactate LDH leakage, restored ATP generation, and ameliorated mitochondrial ultrastructural damage. Mechanistically, harpagide suppressed oxidative stress and alleviated mitochondrial calcium ([Ca^2+^]_mito_) overload, concomitant with modulated expression of MCU complex components and apoptosis-related proteins. These neuroprotective effects were linked to harpagide-mediated AMPK expression and subsequent preservation of mitochondrial bioenergetics and calcium homeostasis. Collectively, these findings position harpagide as a promising phytochemical candidate for ameliorating mitochondrial dysfunction in ischemic stroke, warranting further investigation into its precise molecular interactions with the AMPK–MCU axis.

**Supplementary Information:**

The online version contains supplementary material available at 10.1038/s41598-026-53322-9.

## Introduction

Stroke, a cerebrovascular disorder resulting from cerebral blood vessel rupture or occlusion^1^, is categorized into ischemic and hemorrhagic types. Ischemic stroke comprises 62% of the global stroke cases, and there are over 7.6 million new cases annually^2^. Current reperfusion-based treatments, such as mechanical thrombectomy and recombinant tissue plasminogen activator (rt-PA) thrombolysis, are constrained by factors such as the time window. This limits their applicability to only a fraction of patients ^3,4^. Thus, the development of neuroprotective agents to preserve neural tissue integrity and enhance patient recovery is of paramount urgency.

Harpagide is a major iridoid constituent in *Scrophularia ningpoensis*, and it has been the focus of several recent studies^5^. These studies have indicated in neurological function assessments that harpagide contributes to enhanced scores and a decreased infarct volume in middle cerebral artery occlusion (MCAO) mice by modulating mitochondrial function and inhibiting apoptosis-related pathways^6–8^. The neuroprotective effects of harpagide have been attributed to its ability to inhibit an abnormal increase induced by OGD in the intracellular Ca^2+^ concentration in hippocampal neurons^9^. Although functional benefits have been repeatedly documented, how harpagide exactly exerts these neuroprotective actions still requires a full molecular explanation.

The mitochondrial calcium uniporter (MCU) complex serves as the principal gateway for Ca^2+^ entry into the mitochondrial matrix, playing a pivotal role in cellular bioenergetics. Under physiological conditions, 5'-AMP-activated protein kinase (AMPK)-dependent phosphorylation of MCU at Ser57 enhances channel activity, facilitating moderate Ca^2+^ influx that stimulates ATP synthesis and adapts cellular energy metabolism to metabolic demands^10,11^. However, under pathological conditions such as cerebral ischemia, dysregulated AMPK–MCU signaling drives excessive mitochondrial Ca^2+^ accumulation (mitochondrial Ca^2+^ overload). This pathological Ca^2+^ surge precipitates mitochondrial membrane potential (ΔΨm) collapse, exacerbates ROS production, and gates the opening of the mitochondrial permeability transition pore (mPTP). Consequently, these mitochondrial catastrophes trigger neuronal apoptosis through both cytochrome c/caspase-3-dependent and apoptosis-inducing factor (AIF)/endonuclease G (Endo G)-dependent pathways^12–14^. Despite the critical involvement of the AMPK–MCU axis in ischemic neuronal injury, pharmacological strategies targeting this specific signaling nexus to ameliorate mitochondrial Ca^2+^ dyshomeostasis remain to be fully elucidated.

This study investigates the impact of harpagide on AMPK–MCU signaling, offering preliminary insights into the therapeutic utility of this pathway as a target for ischemic stroke intervention.

## Materials and methods

### Animals

A total of 100 male Institute of Cancer Research (ICR) mice (8 weeks old, 23–25 g, SPF grade) were obtained from Vital River Laboratory Animal Technology Co., Ltd. (Beijing, China). All procedures were approved by the Animal Ethics Committee of Jiaxing University (No. JUMC2022-047) and conducted in accordance with the NIH Guide for the Care and Use of Laboratory Animals. The sample size was predetermined based on our previous studies and pilot experiments to ensure adequate statistical power while accounting for anticipated surgical mortality and occlusion failure rates (approximately 10–15%).

Mice were randomly assigned to sham or middle cerebral artery occlusion (MCAO) surgery using a computer-generated randomization sequence. Intraoperative exclusion criteria included: (1) regional cerebral blood flow reduction < 70% of baseline (measured by laser Doppler flowmetry), (2) intracerebral hemorrhage upon dural inspection, or (3) death during surgery. A total of six animals were excluded—two due to failed occlusion, one due to hemorrhage, and one due to intraoperative death—yielding 96 mice for downstream allocation.

To minimize repeated invasive procedures and ensure data integrity, distinct cohorts were allocated for different endpoints: n = 8 per group for 2,3,5-triphenyltetrazolium chloride (TTC) staining and neurological deficit scoring (six experimental groups; total n = 48), n = 4 per group for regional cerebral blood flow (rCBF) monitoring, and n = 3 per group for transmission electron microscopy (TEM) and immunohistochemistry (IHC) analyses (six experimental groups; total n = 18 for each modality). The remaining animals (n = 4) were retained as reserves to replace potential postoperative losses. Detailed animal allocation and exclusion criteria are provided in Supplementary Table 1.

Surgical procedures were performed by a single investigator, while neurological assessments and histological analyses were conducted by a second investigator blinded to group allocation. TEM image acquisition and quantification were performed by a third independent observer to ensure unbiased assessment of mitochondrial ultrastructure.

### Cells

The PC12 rat pheochromocytoma cell line (CVCL_0481; Cat. No. Delf-10283) and the human neuroblastoma cell line SH-SY5Y (CVCL_0019; Cat. No. Delf-10599) were purchased from Hefei Wanwu Biotechnology Co., Ltd. (Hefei, China). To address the species-specific discrepancy in the AMPK phosphorylation site on MCU (Ser57 is present in human MCU but not conserved in rat orthologs), we employed a dual-cell strategy: SH-SY5Y cells were specifically utilized for p-MCU(Ser57) immunodetection and human-relevant phosphorylation validation, whereas PC12 cells, characterized by their well-established neuronal-like phenotype and extensive validation in OGD models, served as the primary platform for functional mechanistic studies. Data derived from SH-SY5Y cells were interpreted exclusively as confirmatory evidence for human MCU phosphorylation status, while the main mechanistic conclusions regarding harpagide-mediated mitochondrial protection were drawn from the PC12 system.

### Chemicals and regents

The harpagide (99.6% purity was determined using high performance liquid chromatography (HPLC), and it was purchased from the Shanghai Tongtian Biotechnology Co., Ltd. (Shanghai, China; Catalog No. 23102322)),harpagide was freshly prepared before each experiment by dissolving in normal saline (0.9% NaCl) at a stock concentration of 4 mg/mL. The ruthenium red (RR; purity > 95%, HPLC) was obtained from the Shanghai Aladdin Biochemical Technology Co., Ltd. (Shanghai, China; Catalog No. R100832). Compound C (CC; purity > 99.5%, HPLC) was sourced from GLPBIO (Montclair, CA, USA; Catalog No. GC17243) and was suitable for experimental use (Supplementary Fig. 1).

The following antibodies were used in this study: AMPKα (#2532, Cell Signaling Technology, Danvers, MA, USA; 1:1000), AIF (#4642S, Cell Signaling Technology; 1:1000), Cytochrome c (#11940S, Cell Signaling Technology; 1:1000), MCU (#14997S, Cell Signaling Technology; 1:1000), Caspase-3 (#9662S, Cell Signaling Technology; 1:1000), Endo G (ab76122, Abcam plc, Cambridge, UK; 1:2000), p-MCU (synthesized in-house; 1:1000), β-actin (AC026, ABclonal Technology, Shanghai, China; 1:10000), and horseradish peroxidase (HRP)-goat anti-rabbit (ZB-2301, Zhongshan Jinqiao Biotechnology, Zhongshan, China; 1:10000). Additionally, a polyclonal antibody against MCU/CCDC109A (26312–1-AP, ProteinTech Group, Inc., Chicago, IL, USA; 1:200) was also used.

The following reagents and kits were used: the Annexin V-FITC Apoptosis Detection Kit (C1062M, Shanghai Beyotime Biotechnology, Shanghai, China), an LDH assay kit (Lactate Dehydrogenase, A020-2-2, Nanjing Jiancheng Bioengineering Research Institute, Nanjing, China), a SOD assay kit (superoxide dismutase, A001-3-1, Nanjing Jiancheng Bioengineering Research Institute), an MDA assay kit (malondialdehyde, A003-4-1, Nanjing Jiancheng Bioengineering Research Institute), an ATP assay kit (S0027, Shanghai Beyotime Biotechnology, Shanghai, China), a Rhod-2 AM fluorescent probe (ab142780, Abcam), a 2’,7'-dichlorodihydrofluorescein diacetate (DCF-DA) fluorescent probe (S0033S, Shanghai Beyotime Biotechnology), a JC-1 staining kit (C2006, Shanghai Beyotime Biotechnology), and a triphenyl tetrazolium chloride (TTC) dye (Triphenyltetrazolium Chloride, T8877, Sigma-Aldrich Corporation, St. Louis, MO, USA).

### OGD model

An in vitro oxygen–glucose deprivation (OGD) model was established to simulate ischemic injury as previously described^15,16^.Cells were pre-treated with harpagide at different concentrations (50 μM, 100 μM) 48 h prior to OGD modeling^17,18^. Briefly, cells were washed twice with PBS and then incubated in glucose-free EBSS balanced salt solution. The culture plates were transferred to a hypoxia chamber flushed with a gas mixture of 95% N_2_ and 5% CO_2_ for 10 min to achieve and maintain 0.1% O_2_ concentration (severe hypoxia), with continuous monitoring via the incubator’s internal paramagnetic oxygen sensor. The chamber was sealed and maintained at 37 ± 0.5 °C with 95% humidity for 6 h to mimic ischemic and hypoxic conditions. Control cells were cultured in complete medium (DMEM and 10% FBS) under normoxic conditions (21% O_2_, 5% CO_2_, 37 °C, 95% humidity) in the same incubator type.Oxygen levels were independently verified using an OxySense® fiber-optic oxygen probe before and after each experiment to ensure atmospheric stability.

### Western blot

Protein separation was conducted using sodium dodecyl sulfate–polyacrylamide gel electrophoresis (SDS-PAGE), polyvinylidene fluoride (PVDF) transfer, 5% milk blocking, sequential primary and secondary antibody incubations, electrochemiluminescence ECL (GK10008, GLPBIO) development, and ImageJ 1.51 k (Bethesda, MA, USA) densitometry. All procedures were performed according to the standard western-blot protocols.

### RT-PCR

The total RNA was extracted from cultured cells using the TransZol Up reagent (ET111-01-V2, TransGen Biotech, Beijing, China) according to the manufacturer’s instructions. We quantified the total RNA on a NanoDrop 2000 (Thermo Fisher Scientific, Waltham, MA, USA), reverse-transcribed equal amounts to cDNA using kit RK20433 (ABclonal, China), and ran real-time qPCR on the Realplex2 system (Eppendorf AG, Hamburg, Germany).The primer sequences used for qPCR are listed in Supplementary Table 2.

### Apoptosis assay

Cells were harvested immediately after 6-h OGD, 5 μL Annexin V–FITC and 10 μL propidium iodide (PI) were added to the cell suspension and incubated for 15 min at room temperature in the dark according to the manufacturer’s instructions. Samples were analyzed using a flow cytometer (Becton Dickinson, Franklin Lakes, NJ, USA).Cell populations were gated to distinguish viable cells (Annexin V^−^/PI^−^), early apoptotic cells (Annexin V^+^/PI^−^), late apoptotic cells (Annexin V^+^/PI^+^), and necrotic cells (Annexin V^−^/PI^+^).The apoptotic rate was defined as the sum of early and late apoptotic populations and was used for quantitative analysis. A minimum of 10,000 single cells within the gated population were acquired per sample.

### Lactate dehydrogenase (LDH) activity assay

The reaction mixture was assembled per the manufacturer’s instructions for the LDH assay kit. After incubation at 37 °C in the dark, the optical density (OD) was assessed at 450 nm using a microplate reader (Tecan Group Ltd., Männedorf, Switzerland).

### Superoxide dismutase (SOD) activity assay

Following the OGD injury, the cells were treated according to the manufacturer’s guidelines of the SOD assay kit. The SOD activity was determined for each group based on the measured protein concentration^19,20^.

### Malondialdehyde (MDA) content assay

The cells were lysed on ice for 30 min in 150 μL of a lysis buffer. The lysate was collected and mixed with the reagents as per the manufacturer’s instructions. They were then heated at 95 °C for 40 min. After cooling, the optical density (OD) was measured at 532 nm. The MDA content was determined for each group based on the protein concentrations.

### ATP content assay

The experiment was conducted following the manufacturer’s guidelines for the ATP assay kit. The relative light units (RLU) of each group were measured using a GloMAXTM 20/20 Luminometer (Promega Corporation, Madison, WI, USA).

### Evaluation of caspase 3 activity

Caspase-3 enzymatic activity was assessed using a fluorometric Caspase 3 Activity Assay Kit (Abcam, Cambridge, UK) according to the manufacturer’s protocol. Following OGD/R exposure and/or indicated transfections, cellular lysates (50 μg protein) were incubated with the fluorogenic substrate Ac-DEVD-AMC (200 μM) at 37 °C for 1 h. Fluorescence intensity was measured at excitation/emission wavelengths of 380/460 nm and normalized to the vehicle control group.

### [Ca^2+^]_mito_ measurement

The cells were then incubated with the Rhod-2 AM fluorescent calcium indicator working solution in the dark at 37 °C for 30 min. Each well was replenished with phosphate buffer solution (PBS) and incubated at 37 °C for another 30 min. We then performed cell microscopy with an Olympus inverted fluorescence system. The excitation and emission wavelengths applied during imaging were 552 nm and 581 nm, respectively.

### ROS detection

After the OGD exposure, a 100-μL aliquot of the 2’,7'-dichlorofluorescein diacetate (DCF-DA) working solution was introduced into each well. This was followed by a 30-min incubation period at 37 °C. The subsequent fluorescence observation was conducted on an inverted microscope, with the excitation and emission parameters set to 488 nm and 525 nm, respectively.

### Mitochondrial membrane potential assay

Cells were collected and incubated with JC-1 staining solution at 37 °C for 20 min as previously described^21,22^. After staining, cells were washed twice with PBS and resuspended in 500 μL PBS. Mitochondrial membrane potential was quantitatively analyzed using a BD FACSCalibur flow cytometer with excitation at 488 nm. JC-1 monomers (green fluorescence) were detected in the FL1 channel (530 ± 15 nm), whereas JC-1 aggregates (red fluorescence) were detected in the FL2 channel (585 ± 21 nm). The ratio of red to green fluorescence intensity was calculated as an indicator of mitochondrial membrane potential, and at least 10,000 cells were analyzed per sample.

### Permanent MCAO (pMCAO) model

ICR mice were anesthetized with 4% isoflurane for induction, with body temperature maintained at 36.5–37.5 °C. Anesthesia was subsequently maintained with 2% isoflurane throughout the surgical procedure. A nylon monofilament suture (1622-A4, Beijing Xinnong Technology Co., Ltd., Beijing, China) was inserted via the right common carotid artery and advanced to occlude the left middle cerebral artery (MCA). The suture was maintained in place for 8 h of permanent ischemia without reperfusion, establishing the pMCAO model.The neurological function (Longa)^23,24^ and rCBF were then evaluated. Immediately after surgery, the mice were administered different doses of harpagide (4, 8, and 12 mg/kg)^7^^8^ and the RR (3.75 mg/kg), via tail vein injections. The mice in the sham and model groups were injected with an equivalent volume of normal saline (0.1 mL/10 g) using the same method.Animals were monitored for mortality, body weight, and general condition throughout the 8-h experimental period. No significant differences in these parameters were observed between treatment groups.

### 2,3,5-Triphenyltetrazolium chloride (TTC) staining

The brains were removed and sliced into five coronal sections. The slices were then stained with TTC to evaluate the infarct volumes^25^. Infarct volume (%) = (infarct area / contralateral hemisphere area) × 100.

### Transmission electron microscopy (TEM)

The mice were perfused via the left ventricle with normal saline followed by pre-cooled 4% paraformaldehyde under deep anesthesia. After perfusion, the brains were removed, and the cortical temporal lobe penumbra tissue from the ischemic hemispheres were collected and sectioned into blocks no thicker than 1 mm. These samples were fixed overnight in an electron microscopy fixative and then transferred to 3% glutaraldehyde for further processing at 4 °C. Samples were processed by fixing in 1% osmium tetroxide, dehydrating through a graded series of acetone solutions, and embedding in Epox 812 resin. Ultrathin sections were subsequently stained with uranyl acetate and lead citrate and examined under TEM (HT7800, HITACHI, Tokyo, Japan) to observe the mitochondrial ultrastructures. Images were captured for each tissue block at magnifications of 5000 × , 8000 × , and 20,000 × .

### Immunohistochemistry (IHC)

The brain tissues were collected (see above), and 8 μm-thick cryosections were warmed for 30 min, baked at 37 °C for 15 min, and fixed in methanol for 20 min at room temperature. The samples were washed with PBS, and antigen retrieval was performed by heating in a citrate buffer at 98 °C for 20 min. The endogenous peroxidase activity was quenched using 3% H_2_O_2_, followed by blocking with 10% goat serum for 60 min. Sections were incubated with primary antibodies overnight at 4 °C and then with secondary antibodies and biotinylated secondary antibody conjugates (PV6000, Zhongshan Jinqiao) at 37 °C for 30 min. Staining was visualized with 3,3′-diaminobenzidine (DAB) (DAB-0031, Fujian Maxin Biotechnology Development, Fuzhou, China) and counterstained with hematoxylin.Three random fields of the ischemic cortical temporal lobes were imaged at 100 × and 200 × magnifications using an optical microscope. Enlarged images were digitally magnified from the 200 × images. ImageJ 1.51k software was used to analyze the mean positive expression areas.

#### Data and statistical analysis

Statistical analysis was performed using GraphPad Prism 10 or SPSS 22.0 software by an investigator blinded to group allocation. n represents biological replicates, with technical replicates averaged prior to analysis to ensure data independence. Sample sizes were determined based on retrospective power analysis of preliminary data, achieving > 80% power (α = 0.05) to detect effect sizes of 0.8–1.2 for primary endpoints (infarct volume, neurological scores) with the reported n values (8–12 animals per group for in vivo; 6–8 independent experiments for in vitro). Normality was assessed using the Shapiro–Wilk test. For normally distributed data, one-way ANOVA followed by LSD post-hoc test (homogeneous variances) or Games-Howell test (heterogeneous variances) was used. For non-normally distributed data (e.g., Longa scores), the Kruskal–Wallis H test followed by Dunn’s post-hoc test was applied. All values are expressed as mean ± SD. Statistical significance was set at *p* < 0.05 (two-tailed).

## Results

### Harpagide attenuated neurological injury due to cerebral ischemia

**Fig. 1:**
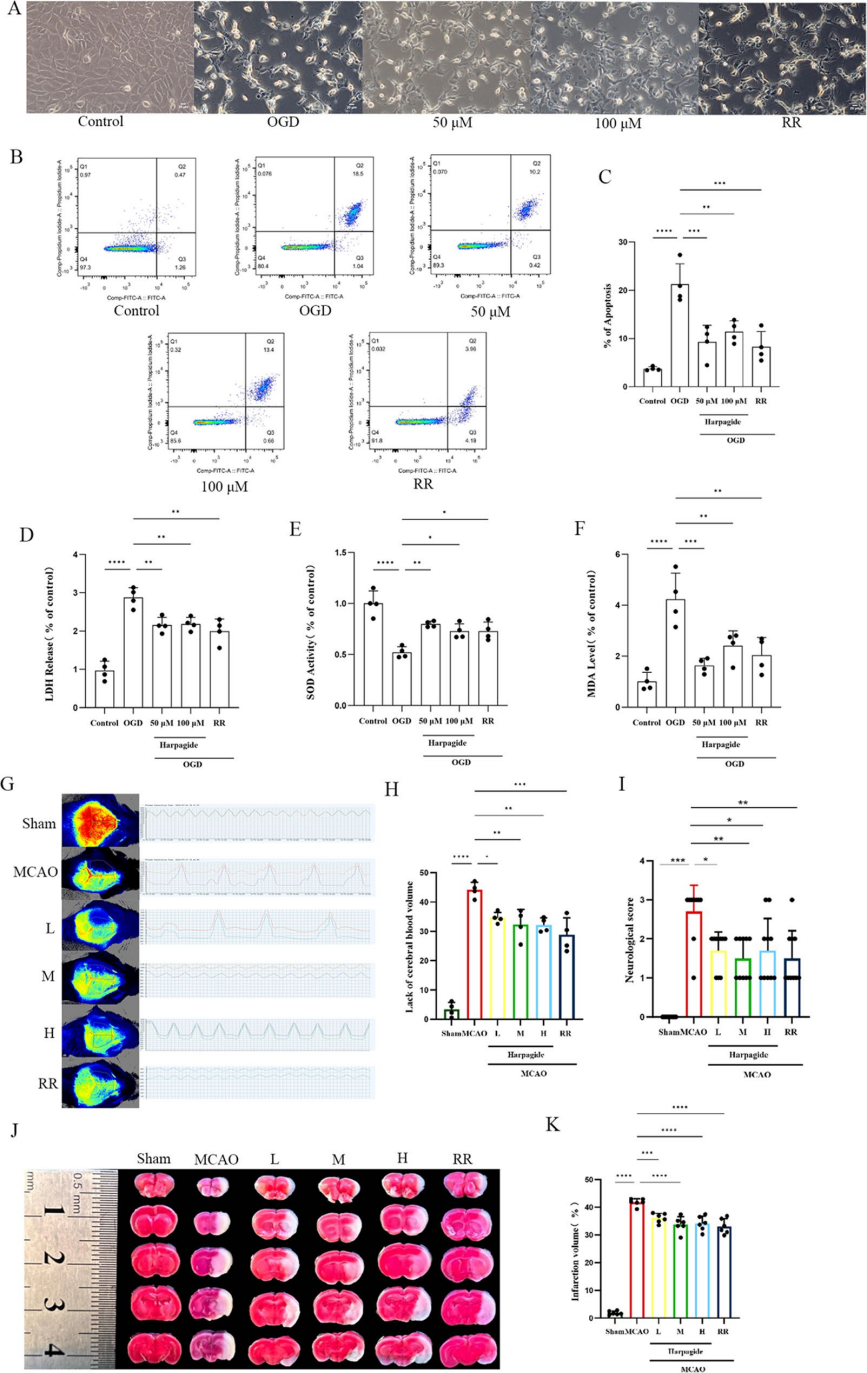
Harpagide attenuated neurological injury induced by cerebral ischemia. (**A**) Morphological alterations in the PC12 cells observed via inverted microscopy; (**B**, **C**) Evaluation of the protective effect of harpagide against OGD-induced apoptosis by Annexin V–FITC/PI double staining.The apoptotic rate was calculated as the total percentage of early apoptotic (Annexin V^+^/PI^−^) and late apoptotic (Annexin V^+^/PI^+^) cells, quantified from the flow cytometry distributions presented in panel B. (**D**) LDH; (**E**) SOD; (**F**) MDA; (**G**–**H**) representative laser speckle images showing that a lack of cerebral blood volume increased in the MCAO animal model; (**I**) Longa neurological function; and (**J**–**K**) representative images of the TTC staining and quantification of the infarct volumes at 8 h after MCAO. Treatment groups were defined as follows: (**L**) MCAO + harpagide 4 mg/kg; (**M**) MCAO + harpagide 8 mg/kg; (**H**) MCAO + harpagide 12 mg/kg; RR, MCAO + ruthenium red (3.75 mg/kg), used as positive control. The Longa neurological score was assessed using the Kruskal–Wallis test, whereas other data were analyzed using a one-way ANOVA. Data are presented as mean ± SD, *p < 0.05; **p < 0.01; ***p < 0.001; ****p < 0.0001.

The effects of harpagide on PC12 cell morphology and apoptosis following 6 h of OGD were evaluated.Pretreatment with 50 or 100 μM harpagide for 48 h appeared to improve cell morphology and density compared to OGD-treated cells (Fig. 1A).Quantitative assessment of cell viability was performed using apoptosis assays (Fig. 1B, C) and LDH release (Fig. 1D).Apoptosis rates were significantly higher in the OGD group compared with the control group (F(4,15) = 18.68, *p* < 0.0001; Fig. 1B, C), whereas treatment with 50 and 100 μM harpagide significantly reduced apoptosis (*p* = 0.0004 and *p* = 0.0025, respectively), suggesting a protective effect against OGD-induced cell death.

The effects of harpagide on OGD-induced cell injury were assessed in this study using various biochemical markers. LDH, a key enzyme in anaerobic glycolysis and gluconeogenesis, is closely linked to cell injury and membrane permeability^26,27^. OGD significantly increased LDH release (F(4,15) = 31.69, *p* < 0.0001), an effect attenuated by 50 and 100 μM harpagide (*p* = 0.0064 and 0.0089, respectively) (Fig. 1D). SOD and MDA are key oxidative stress markers; SOD scavenges ROS to protect mitochondrial integrity^28,29^, while MDA indicates lipid peroxidation-induced injury. OGD injury significantly reduced SOD activity (F(4,15) = 18.34, *p* < 0.0001), an effect reversed by 50 and 100 μM harpagide (*p* = 0.0016 and 0.0174, respectively) (Fig. 1E). OGD also significantly elevated MDA levels (F(4,15) = 14.27, *p* < 0.0001), which were attenuated by 50 and 100 μM harpagide (*p* = 0.0003 and 0.0087, respectively) (Fig. 1F). These findings suggest that harpagide protects PC12 cells against oxidative damage by mitigating lipid peroxidation.

We next evaluated the neuroprotective potential of harpagide in a mouse model of ischemic stroke induced by 8 h of pMCAO, which recapitulates key features of clinical left-hemispheric ischemia. Successful model induction and CBF dynamics were verified by laser speckle contrast imaging and neurological scoring (Fig. 1G, H). Following ischemia, the MCAO group showed a significant reduction in CBF in the affected hemisphere compared with sham controls (F(5, 18) = 54.59, *p* < 0.0001). In contrast, treatment with harpagide (4, 8, and 12 mg/kg) restored CBF in a dose-responsive manner, with all doses reaching statistical significance (*p* = 0.0207, *p* = 0.0034, and *p* = 0.0030, respectively). Consistent with these findings, harpagide administration improved neurological function as assessed by the Longa score across all dosage groups (*p* = 0.034, *p* = 0.008, and *p* = 0.025; Fig. 1I). Moreover, TTC staining revealed a pronounced increase in infarct volume in the MCAO group relative to sham controls (F(5, 30) = 260.9, *p* < 0.0001; Fig. 1J, K). Notably, harpagide treatment significantly reduced infarct volumes at all doses tested (*p* = 0.0010, *p* < 0.0001, and *p* < 0.0001).

### Harpagide suppressed mitochondria-mediated caspase-dependent and -independent apoptosis

**Fig. 2:**
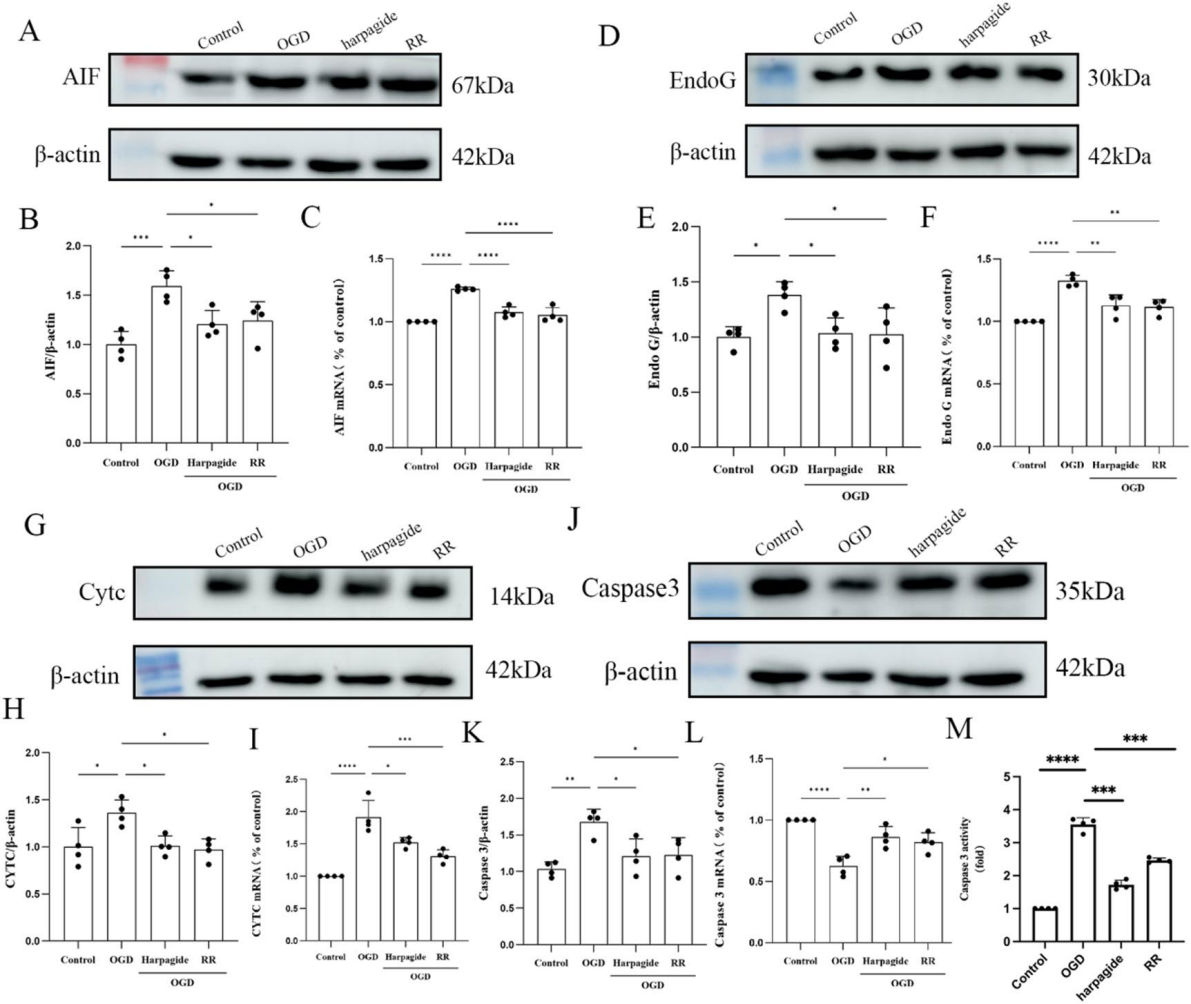
Harpagide suppressed mitochondria-mediated caspase-dependent and -independent apoptosis (**A**) The representative western blot (**B**) quantification of the AIF protein level; (**C**) the AIF mRNA level; (**D**) the representative western blot (**E**): quantification of the EndoG protein level; (**F**) the EndoG mRNA level; (**G**) the representative western blot; (**H**) quantification of the Cyt C protein level; (**I**) the Cyt C mRNA level; (**J**) the representative western blot; (**K**) quantification of the Caspase-3 protein level; (**L**) the Caspase-3 mRNA level; (**M**) Caspase 3 activity of treated PC12 cells as measured by an assay kit. Data are presented as mean ± SD, *p < 0.05; **p < 0.01; ***p < 0.001; ****p < 0.0001.

Mitochondria-mediated apoptotic pathways act synergistically to aggravate ischemic injury. To evaluate whether harpagide inhibits mitochondrial apoptosis, we measured the mRNA and protein expression of AIF, Endo G, caspase-3, and Cyt C in PC12 cells. Western blot analysis (Figs. 2A–K) revealed that the AIF, Endo G, and Cyt C protein levels were significantly elevated in the PC12 cells subjected to OGD compared to controls (F (3, 12) = 9.801, *p* = 0.0009; F (3, 12) = 5.314, *p* = 0.0227; F (3, 12) = 6.424, *p* = 0.0193), whereas the total Caspase-3 protein expression was downregulated (F (3, 12) = 8.028, *p* = 0.0025). Harpagide pretreatment significantly reduced the AIF, Endo G, and Cyt C protein levels (*p* = 0.0205, *p* = 0.0382, *p* = 0.0236) and increased the total Caspase-3 protein expression (*p* = 0.0239). The RT-PCR results (Figs. 2C–L) showed that the AIF, Endo G, and Cyt C mRNA levels were significantly higher in the OGD-treated PC12 cells than those in the controls (F (3, 12) = 35.88, *p* < 0.0001; F (3, 12) = 22.61, *p* < 0.0001; F (3, 12) = 28.31, *p* < 0.0001), while the total Caspase-3 mRNA was downregulated (F (3, 12) = 19.27, *p* < 0.0001). Harpagide significantly decreased the AIF, Endo G, and Cyt C mRNA levels (*p* < 0.0001, *p* = 0.0017, *p* = 0.0112) and increased the total Caspase-3 mRNA (*p* = 0.0023). To functionally validate these findings, we performed fluorometric caspase-3 activity assays using the Ac-DEVD-AMC substrate. Consistent with the immunoblotting data, OGD exposure induced a significant increase in caspase-3 enzymatic activity compared to controls (*p* < 0.001), which was significantly attenuated by harpagide pretreatment. Collectively, harpagide inhibited both the Caspase-dependent and -independent apoptotic pathways mediated by mitochondria, thereby exerting neuroprotective effects against ischemic injury.

### Harpagide mitigated mitochondrial damage triggered by calcium dyshomeostasis post-cerebral ischemia

**Fig. 3:**
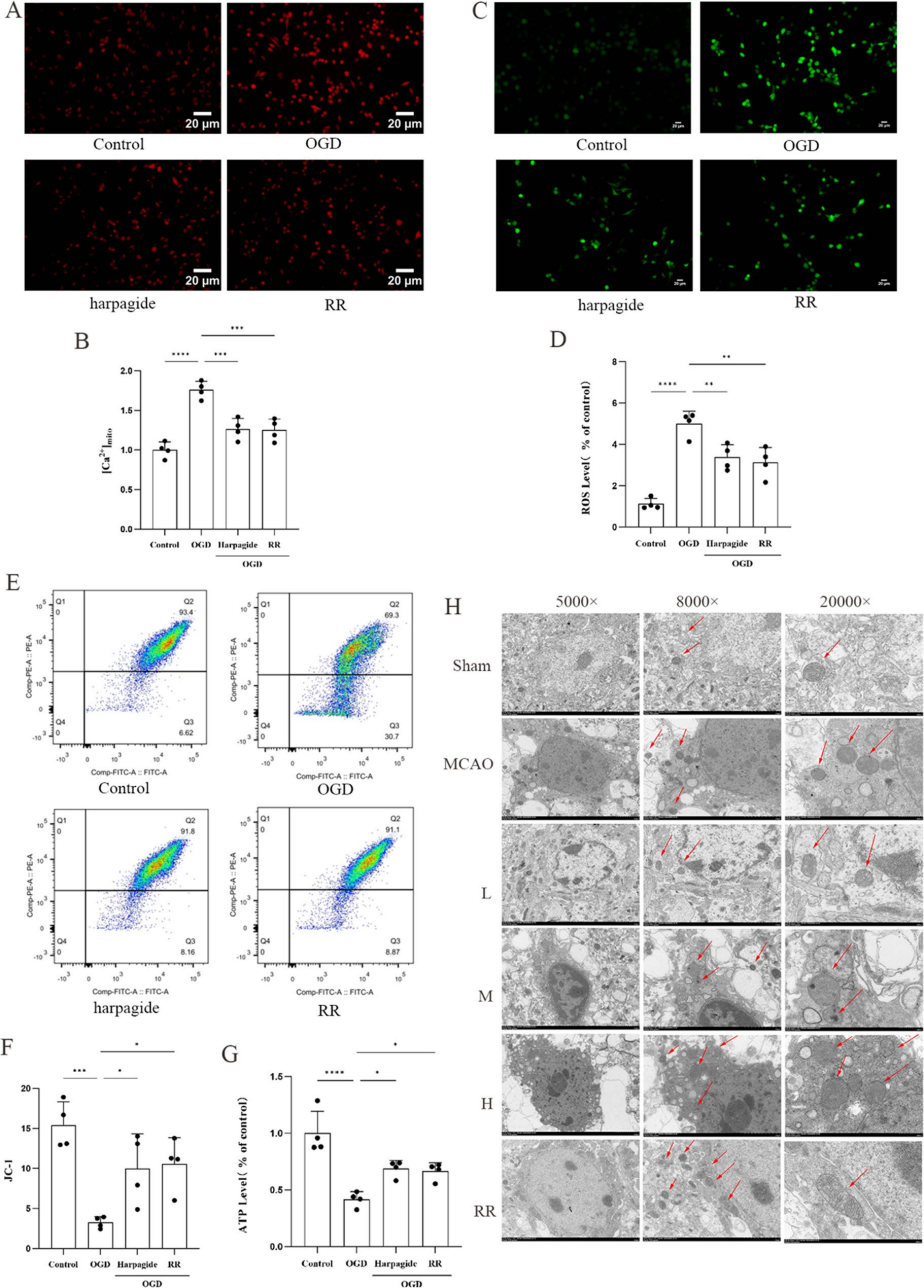
Harpagide mitigated mitochondrial damage triggered by calcium dyshomeostasis post-cerebral ischemia. (**A**) [Ca^2+^]_mito_ in PC12 after OGD of 6 h (200 × , scale bar = 50 μm); (**B**) fluorescence statistics for [Ca^2+^]_mito_; (**C**, **D**) ROS; (**E**–**F**) the JC-1 probe was used to detect the mitochondrial membrane potential (n = 4); G:ATP(n = 4); (**H**) impact of harpagide on the mitochondrial ultrastructure in the neurons of the cortical temporal lobe in the MCAO mice (n = 3). Data are presented as mean ± SD, *p < 0.05; **p < 0.01; ***p < 0.001; ****p < 0.0001.

A fundamental pathological characteristic of cerebral ischemia involves the loss of the calcium equilibrium within the mitochondria^30–32^. To determine whether harpagide modulates mitochondrial calcium dysregulation induced by OGD, we measured the mitochondrial Ca^2+^ concentration ([Ca^2+^]_mito_) in PC12 cells. As shown in Fig. 3A, B, [Ca^2+^]_mito_ was significantly increased in the OGD group compared with controls, confirming mitochondrial calcium overload (F(3, 12) = 27.35, *p* < 0.0001). By contrast, pretreatment with harpagide markedly reduced [Ca^2+^]_mito_ levels relative to the OGD group (*p* = 0.0005), demonstrating its ability to attenuate OGD-induced mitochondrial calcium accumulation.

To further evaluate the protective effect of harpagide on OGD-induced mitochondrial injury in PC12 cells, intracellular ROS (Fig. 3C,D), mitochondrial membrane potential (MMP; Fig. 3E,F) and intracellular ATP content (Fig. 3G) were determined. OGD markedly increased ROS generation (F(3,12) = 30.66, *p* < 0.0001), collapsed MMP (F(3,12) = 10.44, *p* = 0.0006) and depleted ATP (F(3,12) = 17.09, *p* < 0.0001). Harpagide significantly attenuated ROS accumulation (*p* = 0.0081), restored MMP (*p* = 0.0418) and replenished cellular ATP (*p* = 0.0206), underscoring its ability to preserve mitochondrial function.

TEM was employed to visualize the ultrastructural alterations in vivo (Fig. 3F). In the MCAO group, the mitochondria exhibited pronounced vacuolar swelling of the matrix, a marked reduction in electron density, and complete disintegration of the cristae—morphological hallmarks consistent with classical ischemic injury. Compared with the model group, mitochondria in the 4 mg/kg harpagide group retained intact outer membranes but showed disordered and focally absent cristae. The 8 mg/kg dose restored mitochondrial architecture to near normal, with markedly reduced swelling and clearly defined cristae. The 12 mg/kg dose induced minor deformities and sporadic membrane disruption, yet most cristae remained intact. The broad-spectrum calcium channel inhibitor RR produced similar protection: outer membranes were preserved and cristae were clearly visible. Thus, harpagide dose-dependently stabilizes mitochondrial ultrastructure, underpinning its neuroprotective effect.

### Harpagide suppressed MCU expression after cerebral ischemia

**Fig. 4:**
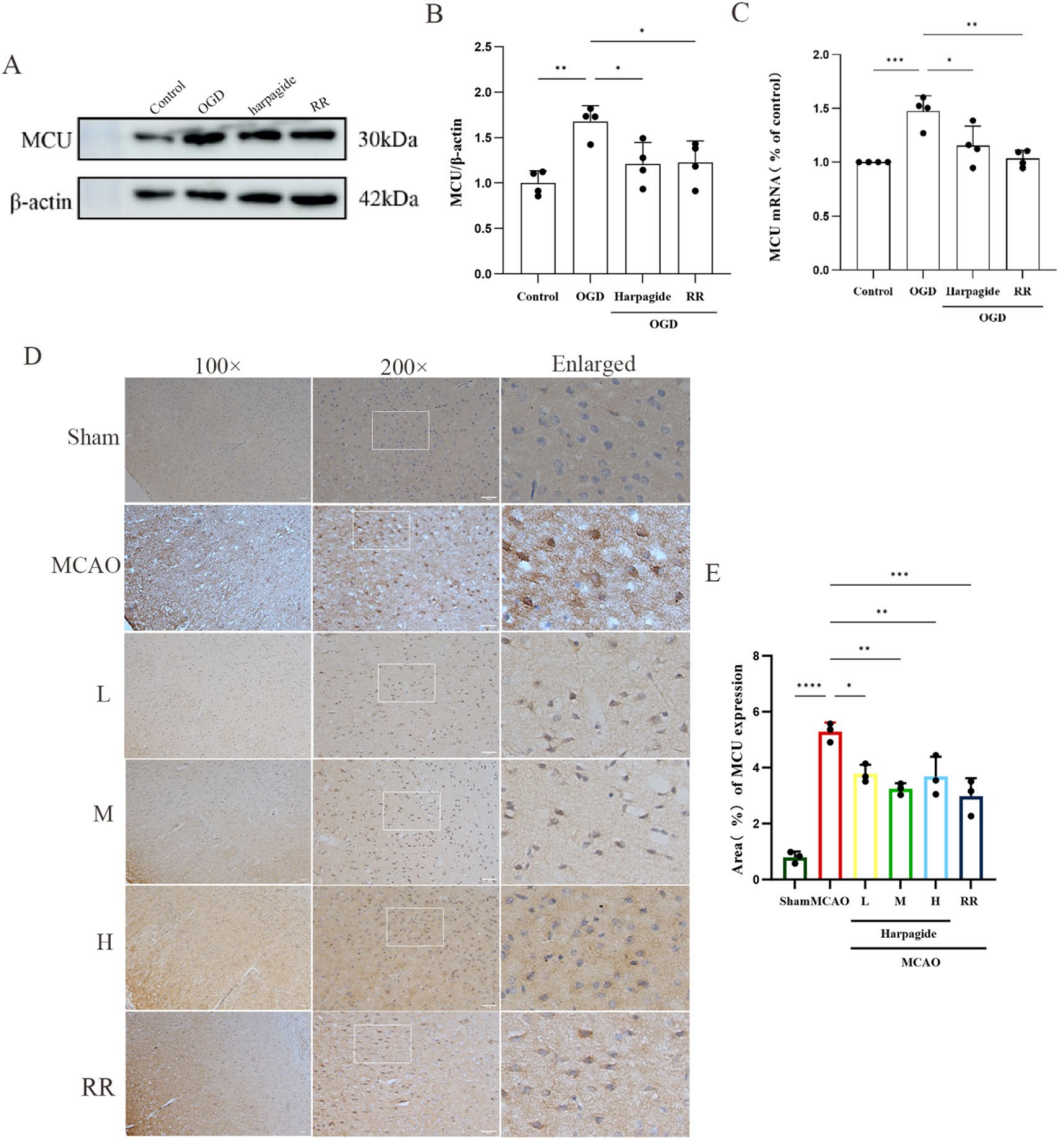
Harpagide suppressed MCU expression after cerebral ischemia. (**A**, **B**) MCU expression was detected using western blot analysis; (**C**) MCU mRNA expression; and (**D**–**E**) immunohistochemical detection of MCU expression in ICR mice brains. Representative images at 100 × and 200 × magnifications are shown, and the enlarged images are digitally magnified views of the boxed regions in the 200 × images. Scale bars: 100 μm (100 ×) and 50 μm (200 × and enlarged images). Data are presented as mean ± SD, *p < 0.05; **p < 0.01; ***p < 0.001; ****p < 0.0001.

The MCU gene and protein expression were then measured to assess the capacity of harpagide to inhibit MCU-mediated mitochondrial calcium overload. A western blot analysis (Fig. 4A,B) showed that the MCU protein expression was significantly higher in the PC12 cells subjected to OGD than those in the controls (F (3, 12) = 8.194, *p* = 0.0021). Harpagide pretreatment significantly reduced the MCU expression (*p* = 0.0278). The RT-PCR results (Fig. 4C) indicated that the MCU mRNA levels were significantly elevated in the OGD-treated PC12 cells compared to the control cells (F (3, 12) = 12.65, *p* = 0.0007). This result was corroborated by the significant MCU mRNA expression reduction due to harpagide (*p* = 0.0131). A marked MCU expression upregulation was detected in the cerebral tissues of the MCAO model compared to the sham group, as demonstrated by immunohistochemical staining (Fig. 4G,H) (F (5, 12) = 31.63, *p* < 0.0001), with vacuolar formation indicative of cell injury. This observation confirmed MCU upregulation following MCAO. Harpagide dose-dependently restricted the MCU-positive field: 4, 8 and 12 mg/kg (i.p.) decreased labelling by 28%, 51% and 44%, respectively (*p* = 0.0145, 0.0013 and 0.0093), with the 8 mg/kg dose producing the maximal suppression and the modest attenuation at 12 mg/kg suggesting an optimal therapeutic window. Collectively, these findings suggested that harpagide alleviated mitochondrial calcium overload and inhibited both caspase-dependent and -independent apoptotic pathways by suppressing MCU expression.

### Harpagide attenuates MCU expression and mitochondrial calcium overload, associated with reduced AMPK expression

**Fig. 5:**
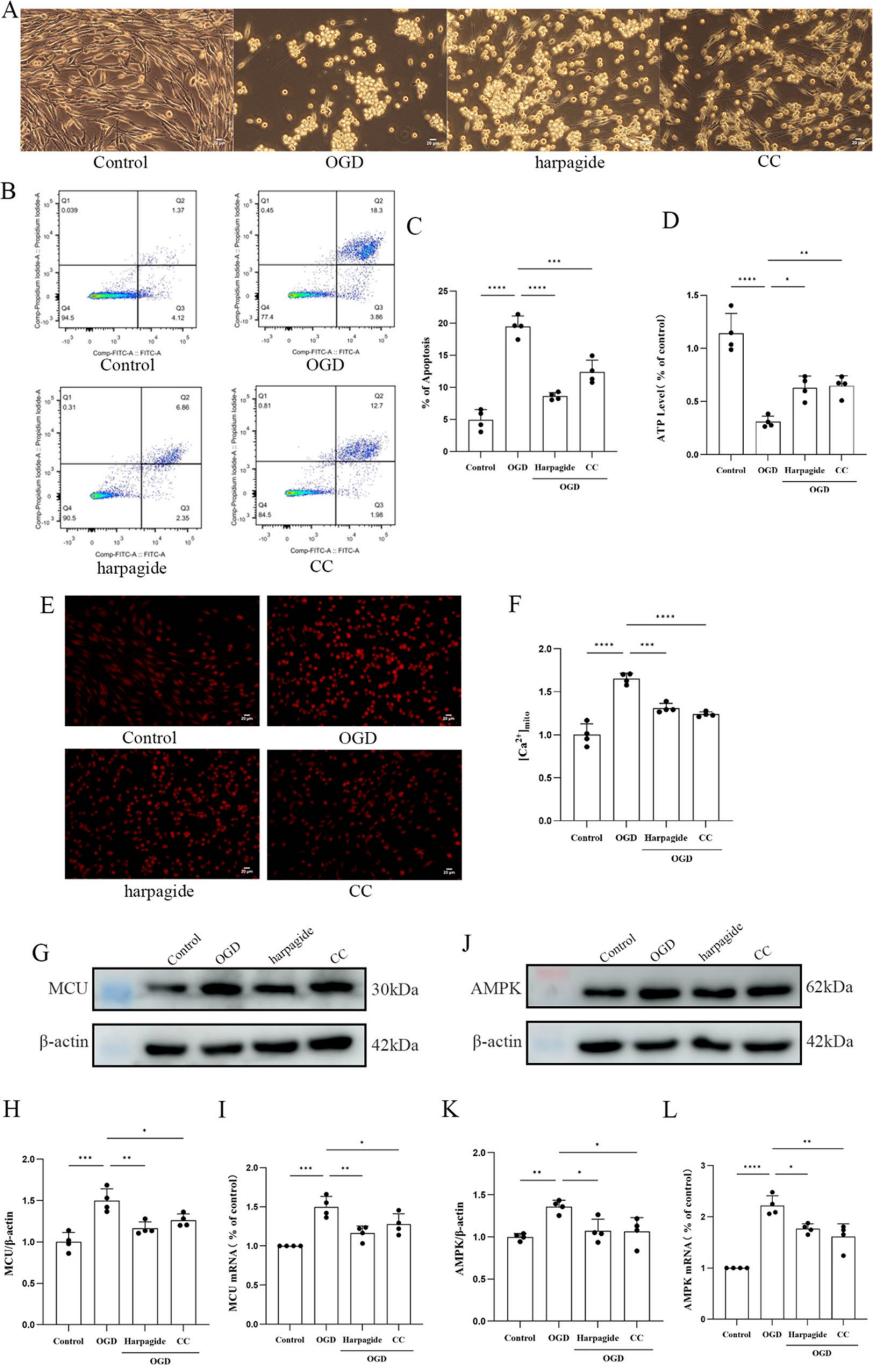
Harpagide attenuates MCU expression and mitochondrial calcium overload, associated with reduced AMPK expression. (**A**) Morphology of the PC12 cells; (**B**, **C**) assessment of the harpagide-mediated reversal of OGD-induced apoptosis via Annexin V-FITC/PI double staining; (**D**) ATP; (**E**–**F**) [Ca^2+^]_mito_; (**G**) the representative western blot; (**H**) quantification of the MCU protein level; (**I**) the MCU mRNA level; (**J**) the representative western blot; (**K**) quantification of the AMPK protein level; and (**L**) the AMPK mRNA level. *p < 0.05; **p < 0.01; ***p < 0.001; ****p < 0.0001 (n = 4,mean ± SD).

We subsequently investigated whether harpagide could mitigate mitochondrial calcium overload through the MCU-AMPK axis. Harpagide reversed OGD-induced reductions in cells and synapses and alleviated cell damage (Fig. 5A). The Annexin V-FITC apoptosis assay showed a significant increase in the apoptosis rate in the OGD-treated PC12 cells (F (3, 12) = 69.49, *p* < 0.0001) that was significantly reduced by the harpagide (*p* < 0.0001). The ATP content assay (Fig. 5D) revealed significantly reduced ATP levels in the OGD group compared to the controls (F (3, 12) = 32.60, *p* < 0.0001), and the harpagide significantly increased the ATP contents (*p* = 0.0125). We next examined whether harpagide attenuates OGD-evoked mitochondrial Ca^2+^ overload via the MCU-AMPK axis. Quantitative imaging revealed a sharp rise in matrix Ca^2+^ fluorescence in the OGD group (F(3,12) = 50.10, *p* < 0.0001); this overload was markedly reduced by harpagide (*p* = 0.0002), concomitant with changes in MCU and AMPK expression. Western blot analysis (Fig. 5G, J) showed significantly elevated MCU and AMPK protein expression in the OGD group compared to the controls (F (3, 12) = 15.74, *p* = 0.0001; F (3, 12) = 7.696, *p* = 0.0041), and the harpagide significantly reduced this expression (*p* = 0.0035, *p* = 0.0191). Similarly, the RT-PCR results (Figs. 5I, L) indicated significantly higher MCU and AMPK mRNA expression in the OGD group (F (3, 12) = 15.84, *p* = 0.0001; F (3, 12) = 36.89, *p* < 0.0001) than in the control group, which was significantly reduced by harpagide (*p* = 0.0035, *p* = 0.0109). Together, these findings indicate that harpagide-induced neuroprotection is associated with reduced MCU expression and improved mitochondrial calcium balance, paralleled by downregulation of AMPK expression.

### Harpagide alleviates mitochondrial calcium overload, accompanied by decreased MCU phosphorylation and AMPK expression

**Fig. 6:**
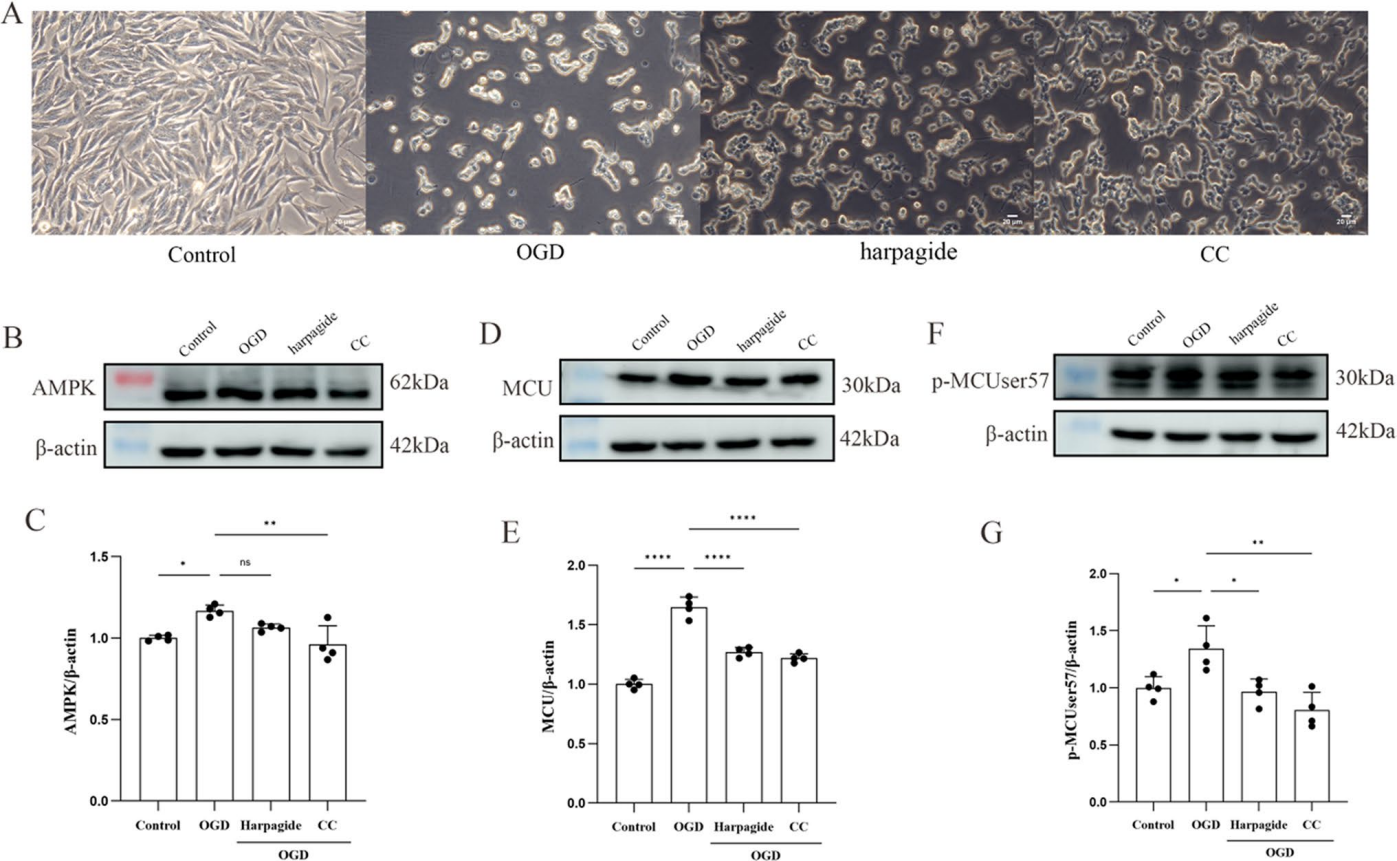
Harpagide alleviates mitochondrial calcium overload, accompanied by decreased MCU phosphorylation and AMPK expression. (**A**) Morphology of the SH-SY5Y cells and the representative western blot and quantification of AMPK (**B**, **C**); MCU (**D**, **E**); and p-MCUser57 (**F**, **G**). *p < 0.05; **p < 0.01; ***p < 0.001; ****p < 0.0001 (n = 4, mean ± SD).

**Fig. 7:**
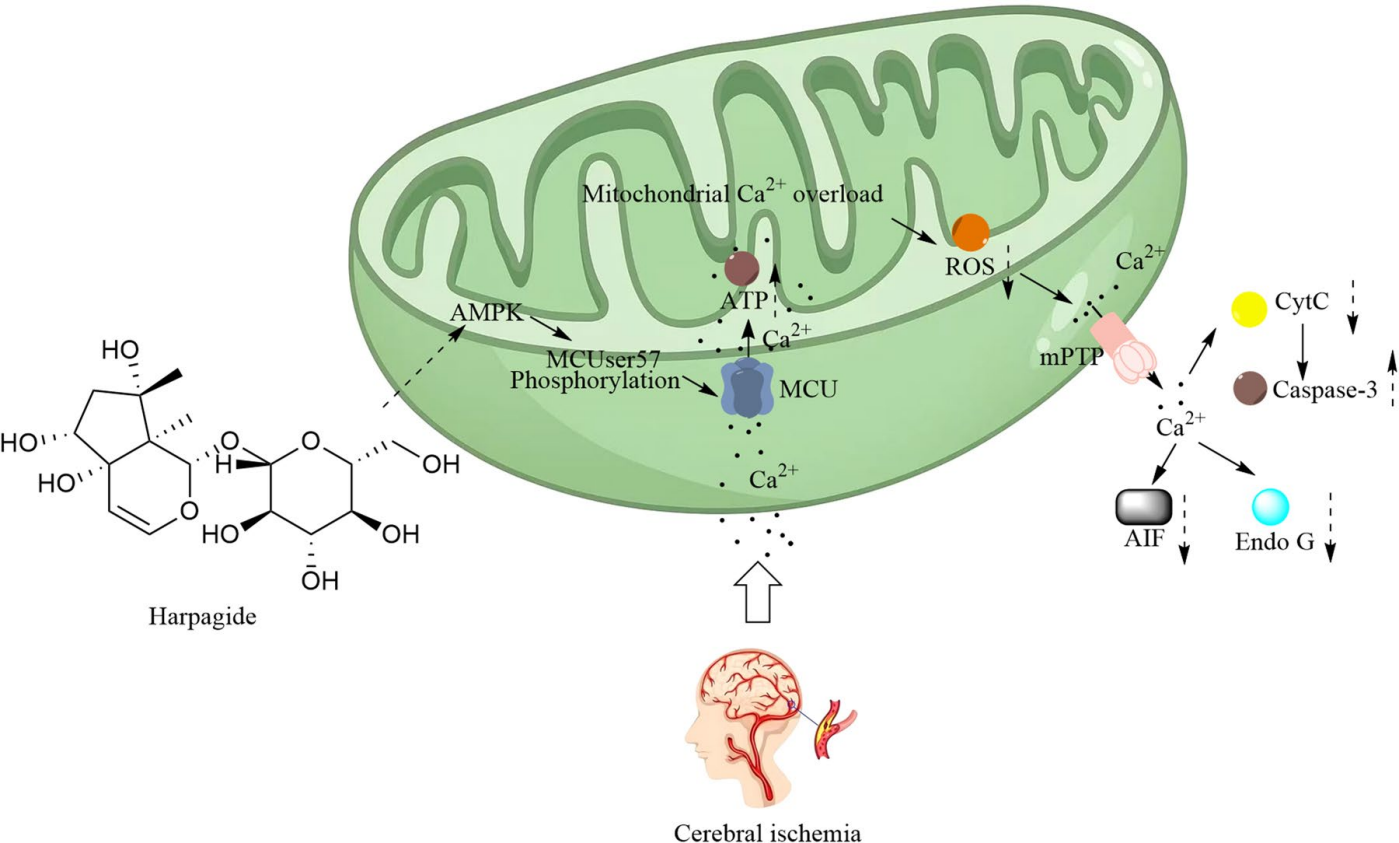
Schematic diagram illustrating the potential mechanism by which harpagide modulates mitochondrial calcium homeostasis in ischemic stroke.

Recent study has further demonstrated that AMPK kinase activity directly governs phosphorylation of MCU at serine 57^10^. Hence, we explored whether harpagide could reduce MCU Ser57 phosphorylation and mitochondrial calcium overload, and examined the relationship to AMPK signaling markers. MCU serine 57 phosphorylation is genome-encoded in humans; therefore, we utilized SH-SY5Y human neuroblastoma cells. Assessment of SH‑SY5Y cell morphology following OGD showed that pretreatment with harpagide for 48 h reversed OGD‑induced reductions in cellular refractivity and in the number of cells and synapses, while also maintaining cytoskeletal integrity (Fig. 6A). Western blot analysis (Fig. 6B–G) revealed that the OGD group exhibited significantly elevated levels of AMPK, MCU, and p-MCU(Ser57) proteins compared to controls (F (3, 12) = 8.716, *p* = 0.0102; F (3, 12) = 96.92, *p* < 0.0001; F (3, 12) = 9.410, *p* = 0.0287). Conversely, harpagide treatment lowered these protein levels relative to the OGD group (*p* = 0.1336, *p* < 0.0001, *p* = 0.0164), with the reduction in p-MCU(Ser57) occurring concomitant with decreased AMPK protein expression. These results indicate that harpagide treatment is associated with reduced MCU Ser57 phosphorylation and attenuated AMPK expression levels under ischemic conditions (Fig. 7).

## Discussion

Cerebral ischemia is a complex disorder of the central nervous system that is marked by inadequate local blood perfusion. This process results in impaired neuronal oxygenation and a lack of metabolic substrates that sets off a cascade of pathological reactions^33^. Harpagide is an iridoid derived from *Scrophularia ningpoensis*, and it has been extensively studied for its neuroprotective effects. In this study, administration of harpagide at doses of 4, 8, and 12 mg/kg significantly ameliorated neurological deficits, restored cerebral blood flow, and reduced infarct volumes in a mouse model of ischemic stroke. Then PC12 cells were used to further elucidate harpagide’s neuroprotective mechanisms, harpagide (50 and 100 μM) significantly decreased OGD-induced apoptosis, LDH release, the MDA content, and restored SOD activity, with the optimal effects observed at 50 μM. Thus, 50 μM harpagide was selected for subsequent mechanistic studies (concurrently, we also screened the harpagide concentration in SH-SY5Y cells, as shown in Supplementary Fig. 2.).

Apoptosis is a genetically orchestrated mechanism of cellular elimination activated by discrete physiological or pathological stimuli^34^. Mitochondrial dysfunction, particularly calcium overload, increases mitochondrial membrane permeability and facilitates the release of apoptogenic mediators such as CytC, AIF and Endo G^35–37^. Our study revealed that harpagide significantly reduced the expression of CytC, AIF, Endo G and total caspase-3. Furthermore, functional assays demonstrated that harpagide suppressed caspase-dependent apoptotic execution, as evidenced by significantly reduced caspase-3 enzymatic activity. Concurrently, harpagide significantly alleviated mitochondrial ultrastructural damage in MCAO mice, stabilized MMP, attenuated OGD-induced membrane depolarization, reduced ROS generation, and restored ATP content. Collectively, these findings constitute direct experimental evidence that harpagide exerts neuroprotective effects concomitant with the amelioration of mitochondrial calcium overload and preservation of mitochondrial structural–functional integrity in both caspase-dependent and -independent apoptotic pathways.

Mitochondrial calcium dyshomeostasis represents a hallmark of cerebral ischemic injury^38^. Under physiological conditions, intracellular and extracellular Ca^2+^ concentrations are tightly regulated; however, ischemia-induced hypoxia and compromised blood supply precipitate aberrant Ca^2+^ accumulation, culminating in mitochondrial calcium overload^39,40^. Our findings that harpagide attenuated Rhod-2 fluorescence, restored mitochondrial bioenergetics, and ameliorated oxidative injury suggest that it modulates mitochondrial calcium handling under ischemic conditions.

MCU, the principal Ca^2+^ uniporter of the inner mitochondrial membrane, governs mitochondrial Ca^2+^ uptake, thereby tuning energy metabolism and apoptosis^41^. During cerebral ischemia, MCU-driven electrochemical import of cytosolic Ca^2+^ disrupts organellar Ca^2+^ homeostasis^42,43^. Genetic evidence shows that MCU knockdown in HeLa cells blunts mitochondrial Ca^2+^ accumulation, whereas its overexpression augments the matrix Ca^2+^ pool^44^. Here, immunohistochemistry revealed that harpagide decreased MCU immunoreactivity in the ischemic cortex of MCAO mice and attenuated neuronal damage; Western blot and qPCR further verified that harpagide down-regulated MCU protein and transcript levels in OGD-injured PC12 cells. These observations constitute direct findings regarding harpagide-mediated MCU downregulation. It should be noted although RR treatment mimicked the neuroprotective effects of harpagide in our experiments, these observations support the general importance of mitochondrial calcium homeostasis in ischemic injury rather than establishing specific MCU dependence. Definitive validation of MCU-specific involvement will require future studies employing highly selective MCU inhibitors (e.g., Ru360) or genetic interventions (MCU knockdown/overexpression).

AMPK acts as a cellular fuel gauge that restores ATP/AMP balance by accelerating glucose and fatty-acid oxidation^45^. Literature indicates that AMPK also fine-tunes mitochondrial Ca^2+^ by phosphorylating Ser-57 of MCU^10,46^. In the present study, harpagide displayed effects comparable to those of the AMPK inhibitor CC, including suppression of PC12 apoptosis and mitochondrial Ca^2+^ overload, restoration of ATP levels, and concomitant down-regulation of AMPK and MCU expression at both the protein and transcript levels. Subsequent experiments in OGD-challenged SH-SY5Y cells demonstrated that both harpagide and CC reduced AMPK and MCU protein abundance, with parallel attenuation of MCU Ser-57 phosphorylation. While these findings demonstrate a consistent correlation between harpagide treatment, reduced AMPK/MCU expression, and attenuated mitochondrial Ca^2+^ overload, they establish an associative rather than causal relationship. Specifically, the observed parallel reduction in AMPK protein levels and MCU Ser-57 phosphorylation indicates that the AMPK–MCU phosphorylation axis is correlatively associated with harpagide’s neuroprotective effects. However, in the absence of direct evidence regarding AMPK enzymatic activity (e.g., phosphorylation status at Thr-172) or genetic rescue/knockout experiments, these data do not permit definitive conclusions regarding the directional causality—whether harpagide acts primarily through AMPK inhibition to subsequently modulate MCU, or whether these molecular changes represent convergent downstream consequences of restored energy homeostasis.

It is critical to delineate the boundaries between the demonstrated findings and the inferred mechanistic framework presented in this study. First, while our data demonstrate that harpagide modulates AMPK-associated signaling to restore mitochondrial calcium homeostasis, the absence of p-AMPK detection precludes definitive conclusions regarding AMPK activation dynamics during ischemic progression. This methodological gap is particularly significant given our preliminary observations that harpagide exerts time-dependent biphasic regulation on mitochondrial calcium dynamics—stabilizing calcium homeostasis during acute ischemic^18^. Furthermore, although harpagide dose-dependently restricted the MCU-positive field, however, we have not established a parallel, dose-dependent gradient in AMPK phosphorylation across these concentrations.Consequently, we position the AMPK–MCU axis as a correlatively associated pathway rather than a proven causal mechanism in the current dataset, future investigations will prioritize harpagide-AMPK interactions as the central mechanistic axis for dissecting anti-ischemic neuroprotection. Second, as Rhod-2 is a non-ratiometric indicator, the present measurements provide relative rather than absolute quantification of mitochondrial Ca^2+^ levels. Future studies employing calibrated or genetically encoded calcium probes will be required for precise quantitative assessment. Third, The present study employed a sustained ischemia paradigm without reperfusion to recapitulate continuous metabolic deprivation and maintain mechanistic alignment with the OGD cellular model. While this approach facilitates focused investigation of mitochondrial failure during persistent ischemic insults, it does not address the effects of post-treatment administration, the therapeutic time window, or long-term outcomes. These critical parameters will constitute essential directions for our future research. Fourth, Immunostaining was performed only with MCU monochromatic staining, which does not allow identification of neurons/Glial cells.

Despite these constraints regarding mechanistic causality, the present findings provide integrated in vivo and i*n vitro* evidence that harpagide preserves mitochondrial integrity, mitigates calcium-associated mitochondrial stress, and attenuates neuronal injury under ischemic conditions. These correlative observations identify mitochondrial calcium regulation as a potential contributor to the neuroprotective profile of harpagide and support further mechanistic and translational investigation to establish definitive causal relationships.

## Conclusion

Collectively, our findings demonstrate that harpagide confers significant neuroprotection against cerebral ischemic injury. Direct experimental evidence indicates that this compound exerts its effects through multiple observed mechanisms, including attenuation of oxidative stress, reduction of mitochondrial calcium overload, enhancement of mitochondrial bioenergetic function, and suppression of mitochondria-driven apoptotic pathways. In vivo experiments further established that harpagide administration dose-dependently reduced cerebral infarct volume, ameliorated neurological deficits, limited ischemic damage, preserved mitochondrial ultrastructure, and decreased MCU immunoreactivity. Whereas our previously study reported that harpagide protects against ischemic injury via mitochondrial pathway^7^, the present study provides novel correlational evidence identifying the AMPK–MCU signaling axis as a potential mechanistic contributor to harpagide-mediated restoration of mitochondrial calcium homeostasis, thereby completing the transition from phenomenological observation to mechanistic pathway elucidation.

## Supplementary Information

Below is the link to the electronic supplementary material.

Supplementary Material 1

Supplementary Material 2

## Data Availability

The data that support the findings of this study are available from the corresponding author upon reasonable request.
