# Supplementary material for: Harpagide attenuates cerebral ischemic injury by modulating mitochondrial calcium homeostasis associated with the AMPK–MCU axis
Source: Sci Rep. 2026 Jun 1;16:25022. doi: 10.1038/s41598-026-53322-9 (PMC13462545; doi:10.1038/s41598-026-53322-9)
Supplement: Supplementary file 1 [file 41598_2026_53322_MOESM1_ESM.docx]

**Figur 2A	Figur 2D**

harpagide

harpagide

Control

Control

RR

OGD

RR

OGD

kDa

kDa

42

30

Endo G

Actin

42

67

Actin

AIF

30

23

18

75

55

75

55

42

42

**Figur 2G	Figur 2J**

75

55

42

30

23

18

kDa

42

35

Actin

Caspase3

Control

200

140110

75

55

42

30

23

18

10

42

harpagide

harpagide

RR

OGD

Control

RR

OGD

kDa

14

Actin

Cytc

**Figur 4A	Figur 5G**

42

30

23

42

30

23

Control

CC

OGD

kDa

30

Actin

MCU

42

harpagide

42

harpagide

Control

RR

OGD

kDa

30

Actin

MCU

**Figur 5J	Figur 6B**

Control

Control

OGD

CC

CC

OGD

55

42

200

140110

75

42

110

75

55

62

Actin

AMPK

kDa

62

Actin

AMPK

42

harpagide

42

harpagide

kDa

**Figur 6D	Figur 6F**

Control

OGD

CC

55

42

30

Actin

MCU

42

harpagide

kDa

30

23

18

55

42

30

23

18

Actin

p-MCUser57

Control

OGD

harpagide

CC

kDa

42

30
