# Supplementary material for: Harpagide attenuates cerebral ischemic injury by modulating mitochondrial calcium homeostasis associated with the AMPK–MCU axis
Source: Sci Rep. 2026 Jun 1;16:25022. doi: 10.1038/s41598-026-53322-9 (PMC13462545; doi:10.1038/s41598-026-53322-9)
Supplement: Supplementary file 2 [file 41598_2026_53322_MOESM2_ESM.docx]

**Harpagide from *Scrophularia*: Regulation of AMPK-MCU Phosphorylation to Inhibit Mitochondrial Calcium Overload and Alleviate Cerebral Ischemic Injury**

Ke Wang ^a,1,*^ Huai-yu Liu ^b,1^ Yue-Wang ^c^ Gen-zheng Su ^d^ Jin-lai Gao ^a^ Yan-Ping Wang ^e^ Zhi He ^a,b,*^

^a^ Department of Pharmacology, College of Medicine, Jiaxing University, Jiaxing, 314001, PR China.

^b^ College of Medicine and Health Sciences, China Three Gorges University, Yichang, 443002, PR China.

^c^ Xinmai Pharmaceutical Co., Ltd. Beijing, 100195,PR China

^d^ College of Life Science and Medicine, Zhejiang Sci-Tech University, Hangzhou, 310018, PR China

^e^ Second Affiliated Hospital of Jiaxing University, Jiaxing, 314001, PR China.^*^ Corresponding author.

E-mail address: wangke@zjxu.edu.cn(K. Wang), lhuaiyu1999@163.com(Y. Liu), 17381350209@163.com(Y. Wang), 2023220903020@mails.zstu.edu.cn(G.Su), [2300758172@qq.com(Y](mailto:2300758172@qq.com(Y). Dong), yueyue.cc11@foxmail.com(Y. Chen), [ypwang93@163.com(Y](mailto:ypwang93@163.com(Y). Wang), hezhi2023@zjxu.edu.cn(Z. He)

^1^ These authors contributed equally to this work

**Supplemental Data**

**Table of Contents**

[Figure S1. Quantitative Reference Material of Harpagide.	2](#_Toc13378)

Figure S2 Dose-Dependent Protective Effects of Harpagide against OGD-Induced Injury in SH-SY5Y Cells……………………………………………………………………………………..3

Table S1 Animal allocation and exclusion criteria	[4](#_Toc7609)

Table S2 Primer Sequences for RT-PCR Analysis	[4](#_Toc14975)

**Quantitative Reference Material of Harpagide**

The structural formula of harpagide, drafted using Chemdraw 20.0 (Cambridge, MA, USA) software, is shown in Figure S1A. The structural formula of Ruthenlum Red and Compound C are presented in Figure S1B-C. The harpagide content was determined using high-performance liquid chromatography (HPLC), and it was 99.6%.

Figure S1. Quantitative Reference Material of Harpagide. (A-C) The structural formula of harpagide, Ruthenlum Red and Compound C. (D) HPLC of harpagide.

Figure S2. Dose-Dependent Protective Effects of Harpagide against OGD-Induced Injury in SH-SY5Y Cells. The effect of harpagide on the SH-SY5Y cells was assessed with an MTT assay. Harpagide showed little or no effect on cell viability at a concentration of 10 μΜ. However, compared to the OGD group, at harpagide concentrations of 10 and 100 μΜ, the cell viability significantly increased. The cell viability further increased at a concentration of 150 μΜ. The data are expressed as the percentage of total cells and are presented as the mean ± SD(n=3). ^**^P < 0.01, significantly different from sham group. ^#^P < 0.05, significantly different from OGD group.

# Table S1 Animal allocation and exclusion criteria

| Group | N (TTC/Longa/rCBF) | N (TEM) | N (IHC) | Subtotal |
| --- | --- | --- | --- | --- |
| Sham | 10 | 3 | 3 | 16 |
| MCAO | 10 | 3 | 3 | 16 |
| MCAO + Harpagide 4 mg/kg | 10 | 3 | 3 | 16 |
| MCAO + Harpagide 8 mg/kg | 10 | 3 | 3 | 16 |
| MCAO + Harpagide 12 mg/kg | 10 | 3 | 3 | 16 |
| MCAO + RR 3.75 mg/kg | 10 | 3 | 3 | 16 |
| Failed occlusion | 2 | - | - | 2 |
| Hemorrhage | 1 | - | - | 1 |
| Death | 1 | - | - | 1 |
| **Total** | **64** | **18** | **18** | **100** |

# Table S2 Primer Sequences for RT-PCR Analysis

| **Gene Target** | **Primer Sequence (5'-3')** |
| --- | --- |
| MCU | Forward: AACGCTGCCAGTTCACACTCAAG  Reverse: AAGTCATCGAGGAGCAGGAGGTC |
| AMPK | Forward: ATGATGAGGTGGTGGAGCAGAGG  Reverse: GGTTCTCGGCTGTGCTGGAATC |
| AIF | Forward: GCCTGGCGGGTGCTTTCAAG  Reverse: TGCTGGAACAAGTTGCCTGGAAG |
| Cyt c | Forward: CCAGGCTGCTGGATTCTCTTACAC  Reverse: AGGTCTGCCCTTTCTCCCTTCTTC |
| Endo G | Forward:TGGACGACACCTTCTACCTGAGC  Reverse:GCTGCGGCTGATCTTCTCAAGG |
| Casepase-3 | Forward: ACGAACGGACCTGTGGACCTG  Reverse: GTTTCGGCTTTCCAGTCAGACTCC |
| β-actin | Forward: CTGAGAGGGAAATCGTGCGTGAC  Reverse: AGGAAGAGGATGCGGCAGTGG |
